# Oscillometry with or without spirometry for methacholine testing

**DOI:** 10.14814/phy2.70387

**Published:** 2025-05-22

**Authors:** Cyndi Henry, Magali Boucher, Marie‐Ève Boulay, Andréanne Côté, Ynuk Bossé

**Affiliations:** ^1^ Institut Universitaire de Cardiologie et de Pneumologie de Québec (IUCPQ) Université Laval Québec City Québec Canada

**Keywords:** asthma, methacholine testing, oscillometry, reactance, respiratory mechanics, spirometry

## Abstract

Oscillometry is proposed as a complementary technique to spirometry for methacholine testing. Yet, before being prescribed in conjunction with spirometry, the extent by which the oscillometric readouts are influenced by spirometric maneuvers, especially the deep inspirations, will need to be determined. Herein, 16 very mild‐to‐mild asthmatics underwent two methacholine challenges on separate visits. On visit 1, the response was tracked by both oscillometry and spirometry, and the challenge was stopped at the provocative concentration causing a decline in forced expiratory volume in 1 s of at least 20%. The same concentration regimen was used on visit 2, but the response was tracked by oscillometry only. The results demonstrated that, except for resistance at 19 Hz, the changes in all oscillometric readouts were greater in the challenge without spirometry (*p* ≤ 0.02). The maximal change in reactance at 5 Hz (X_rs5_), for example, was on average 75.8% greater in the challenge without than with spirometry (*p* = 0.025). The number of doubling concentrations of methacholine that was needed to reach an equivalent change in X_rs5_ was also lower without concomitant spirometry (*p* = 0.0078). It is concluded that the deep inspirations that are required in spirometry to monitor the response to methacholine decrease the oscillometric response.

## 1 INTRODUCTION

Testing the response to inhaled methacholine is useful in clinical settings for guiding asthma diagnostics (Cockcroft, 2010; Nair et al., 2017; O'Byrne, 2010). The test is conventionally measured by spirometry and, therefore, requires the patient to perform forceful breathing maneuvers throughout the methacholine challenge to track the changes in forced expiratory volume in 1 s (FEV_1_). Alternatively, the methacholine response can be measured by oscillometry (King et al., 2020), a technique currently spreading in clinical settings (Donohue & Kaminsky, 2024; Lundblad et al., 2021). Since oscillometry is effortless, it is often promoted for people unable to perform the forceful spirometric breathing maneuvers. These include children, the elderly, and patients for whom the forced maneuvers are contraindicated (Jordon et al., 2022). Yet, readouts collected by oscillometry are also very different from spirometry (Bossé, 2022). They also better disaggregate the contribution of large versus small airways (Chiu et al., 2020; Cottini et al., 2023). Spirometry and oscillometry are therefore complementary, and it would be worthwhile to use them together in all people during the methacholine challenge.

One inconvenience is that the measurement of FEV_1_ in spirometry requires forceful expiratory maneuvers starting from a full deep inspiration. While the dynamic compression of the forceful expiration does not seem to influence the methacholine response (West et al., 2012), the deep inspiration is known to attenuate it by causing a significant bronchodilation (Allen et al., 2008; Chapman et al., 2011; Duggan et al., 1990; Hida et al., 1984; Kapsali et al., 2000; King et al., 2001; Malmberg et al., 1993; Nadel & Tierney, 1961; Salerno et al., 2005; Scichilone et al., 2001; Skloot & Togias, 2003). Therefore, whether oscillometry should be used in conjunction with spirometry for methacholine testing in clinical settings remains uncertain. The crux lies in the extent to which the oscillometric readouts are influenced by the deep inspirations that are required in spirometry to monitor the response to methacholine, which is specifically addressed herein in asthmatic patients.

## 2 MATERIALS AND METHODS

All procedures were approved by the ethics committee of the IUCPQ (protocol 21,001) and all volunteers gave written informed consent. Patients were recruited from the staff and students of *Université Laval*. Only patients with an asthma severity classified at steps 1–3 of GINA guidelines (Global Initiative for Asthma, n.d.) were included. The other inclusion and exclusion criteria are described in a supplementary file.

Patients were enrolled from January to July 2024. All subjects attended three visits (visits 0, 1 and 2). Each visit was separated by at least 48 h, with an average of 9.3 ± 4.3 days. For medicated asthmatic patients, short‐ and long‐acting β_2_‐agonists were prohibited for at least 8 and 24 h, respectively, before each visit. Inhaled corticosteroids and leukotriene receptor antagonists were also prohibited before testing on each visit day. Baseline lung function measured by spirometry and respiratory mechanics measured by oscillometry were assessed at each visit. Owing to the waxing and waning nature of asthma, these baseline measurements were important to ascertain that the patients' basal states were on average similar and, therefore, unlikely to influence the differences in the methacholine response between visits. For these baseline measurements, spirometry was performed before oscillometry, but at least 10 min separated both techniques in order to avoid the bronchoprotective effect of deep inspirations (Allen et al., 2008; Chapman et al., 2011; Duggan et al., 1990; Hida et al., 1984; Kapsali et al., 2000; King et al., 2001; Malmberg et al., 1993; Nadel & Tierney, 1961; Salerno et al., 2005; Scichilone et al., 2001; Skloot & Togias, 2003). As an indirect index of eosinophilic inflammation, the fraction of exhaled nitric oxide (FeNO) was also measured on each visit.

Lung volumes, including total lung capacity (TLC), residual volume (RV) and functional residual capacity (FRC), were also measured at visit 0 by body plethysmography (Platinum Elite™ body plethysmograph with RTD, MGC Diagnostics Corporation, Saint Paul, MN). Predicted values for spirometric lung function and plethysmographic lung volumes were from Quanjer et al. (Quanjer et al., 2012) and Hall et al. (Hall et al., 2021), respectively.

**FIGURE 1:**
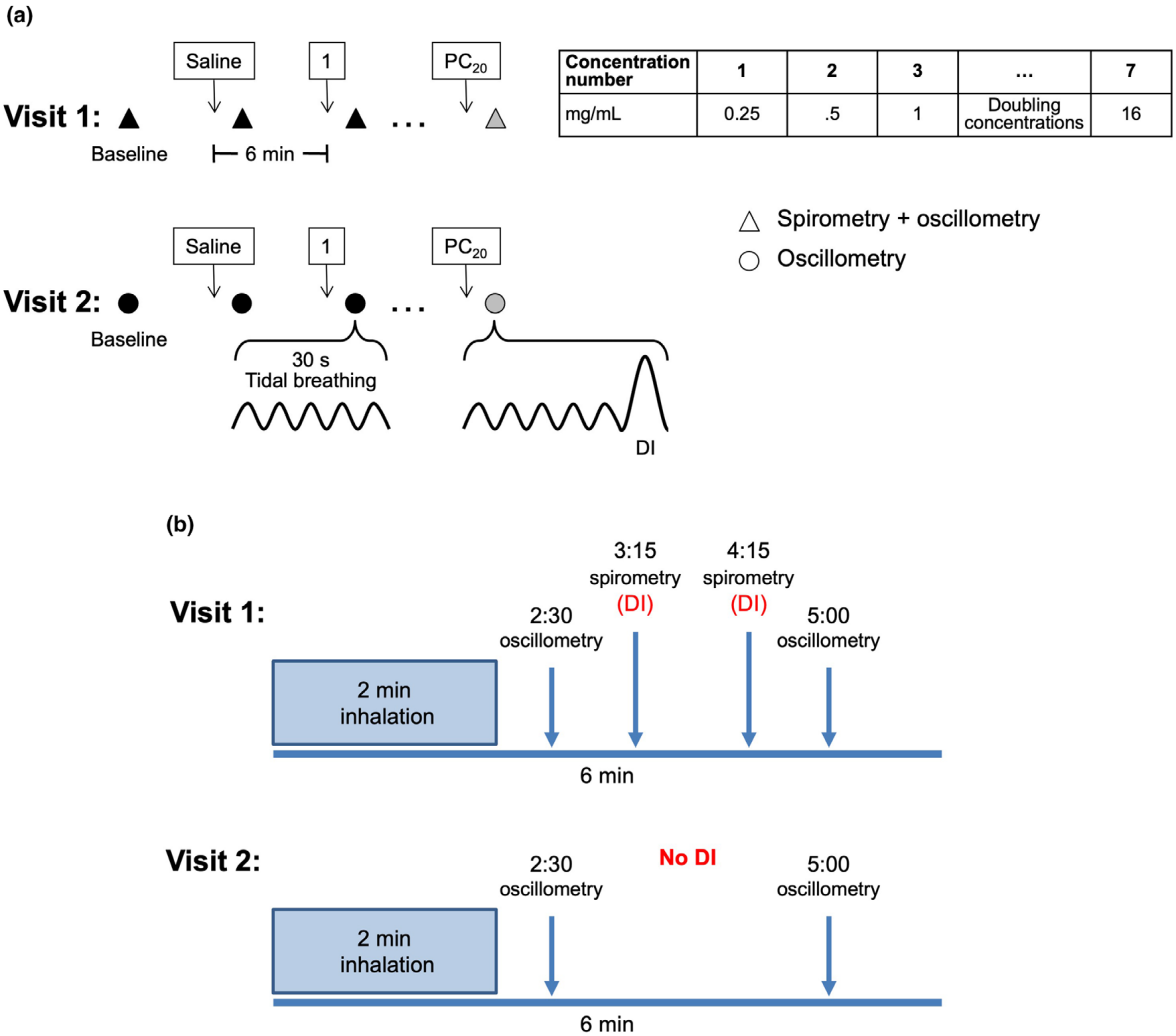
The two methacholine challenges used in this study. The dosing regimens are illustrated in (a), and the sequence of interventions undertaken at each methacholine concentration are presented in (b). At each visit, patients were challenged with incremental concentrations of methacholine (upper right table) at 6 min interval using the 2‐min tidal breathing method. While the parameters of respiratory mechanics were measured by oscillometry at 2.5 and 5 min after the beginning of nebulization of each concentration in both visits, the forced expiratory volume in 1 s (FEV_1_) was additionally measured by spirometry at 3.25 and 4.25 min in visit 1. Triangles indicate where both spirometry and oscillometry were performed, while circles indicate where only oscillometry was performed. The challenge in visit 1 was stopped when the PC_20_ (the concentration causing a decline in FEV_1_ of at least 20%) was reached. The PC_20_ was also used as the final concentration in visit 2. Each oscillometric measurement lasted approximately 30 s (black symbols), over which time the patients were asked to breath normally at tidal volume. The only exception is at the very last oscillometric measurement (at 5 min) after the final concentration (gray symbols), where they were instructed to take a deep inspiration (DI) to total lung capacity after the ~30‐s measurement before leaving the mouthpiece.

Methacholine challenges were performed on visits 1 and 2 using the tidal breathing method (Coates et al., 2017). The procedures are detailed in Figure 1. Most procedures on visit 1 are consistent with current guidelines (Coates et al., 2017). The major difference is that oscillometry was also used to monitor the methacholine response (Figure 1). Oscillometry was performed as recommended by current guidelines (King et al., 2020). These oscillometric measurements also compelled us to extend the time interval between concentrations to 6 min, instead of the 5 min recommended in guidelines (Coates et al., 2017). Another difference with guidelines is that we are using concentrations instead of doses to report the methacholine response. Since this is an interventional study where the same subjects are tested twice, the use of concentrations instead of doses should not have affected the comparison between visits. Yet, it is acknowledged that different doses may have been delivered depending on the output of the nebulizer and the breathing pattern of the participants on each testing day. The methacholine test was stopped when: (1) the concentration of methacholine causing a 20% decline in FEV_1_ (PC_20_) was reached, which was determined in visit 1 but was also used as the final dose in visit 2; (2) the maximal concentration of 16 mg/mL was attained; or (3) the perception of dyspnea rose to 7/10 or above on the Borg scale.

The effect of spirometry on the oscillometric response to methacholine was assessed by comparing the maximal response between visits. Several oscillometric readouts were used to quantify this difference. They include: (1) resistance of the respiratory system at 5 Hz (R_rs5_), an increase reflecting narrowing throughout the entire airway tree; (2) resistance of the respiratory system at 19 Hz (R_rs19_), an increase reflecting large airway narrowing; (3) R_rs5_ minus R_rs19_ (R_rs5‐19_), an increase reflecting small airway narrowing and narrowing heterogeneity; and (4) three different indices of lung stiffness, namely reactance of the respiratory system at 5 Hz (X_rs5_), resonant frequency (F_res_), and reactance area (Ax), which respectively decrease, increase, and increase when the lung becomes stiffer due to tissue stiffening and/or airway closure.

**FIGURE 2:**
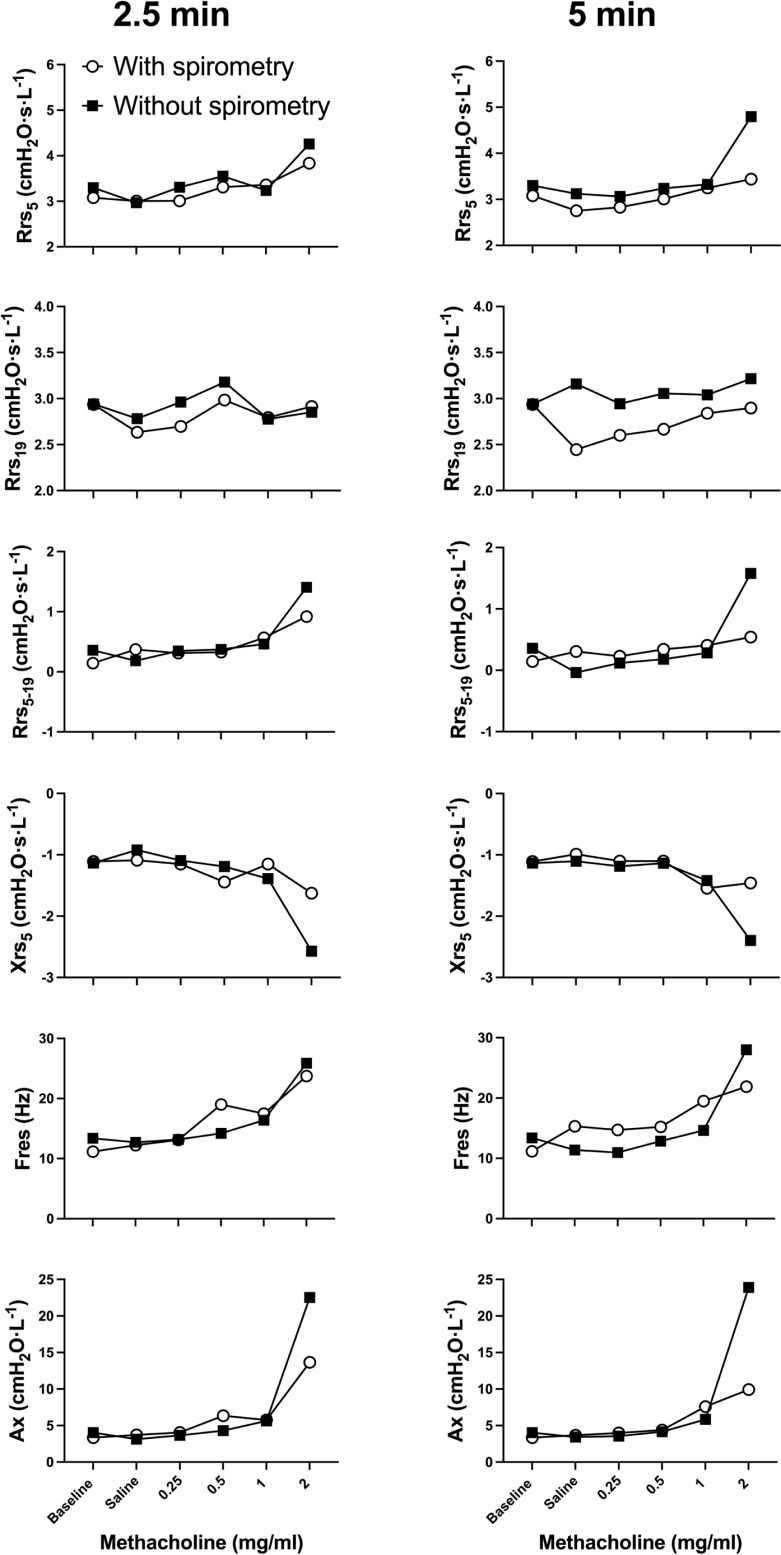
Representative oscillometric responses to methacholine of one patient on visit 1 (open circles), where spirometry was performed, and on visit 2 (solid squares), where spirometry was not performed. The results at 2.5 and 5 min after the beginning of nebulization of each concentration are displayed on the left and right, respectively. As seen on graphs, this patient inhaled four concentrations of methacholine. Respiratory system resistance at 5 Hz (R_rs5_), respiratory system resistance at 19 Hz (R_rs19_), R_rs5_ minus R_rs19_ (R_rs5‐19_), respiratory system reactance at 5 Hz (X_rs5_), resonant frequency (F_res_) and reactance area (Ax) are shown from top to bottom.

The end‐tidal expiratory lung volume (EELV) was also measured at the end of each methacholine challenge. This was done to assess the lung volume at which the subject was breathing at the end of the methacholine challenge and to detect the presence, and quantify the extent, of hyperinflation. More precisely, it was done at the very last oscillometric measurement (at the 5 min time‐point in Figure 2b). After breathing at tidal volume for approximately 30 s, as per all the preceding oscillometric measurement time‐points, the subjects were instructed to take a deep inspiration to TLC before leaving the oscillometric device. The volume trace, which was continuously recorded during the oscillometric measurement, thus contained a maximal point representing TLC. By referring this maximal point to TLC measured at visit 0 by plethysmography, it was possible to calculate the volume at which the subjects were breathing at any time during this last oscillometric measurement. This is valid assuming that TLC is relatively stable over time and not influenced by methacholine. The EELV was calculated by taking the volumes at the end of each tidal expiration and averaging them over the entire period of oscillometric measurement (i.e., ~30 s). Hyperinflation was subsequently calculated by subtracting FRC (measured on visit 0) from EELV, and is expressed in percentage of TLC.

### 2.1 Statistics

Paired t‐tests were used to compare between the two visits. The methacholine response for all oscillometric and spirometric readouts was fitted to a second order polynomial. The curves were then used for interpolating the PC_20_. A Wilcoxon test was used to compare the number of doubling concentrations between visits. Statistical analyses were performed using Prism (version 10.4.0, GraphPad, San Diego, CA) and *p* < 0.05 was considered significant.

## 3 RESULTS

A total of 16 asthmatic patients were recruited, and all completed the study. Demographics and baseline characteristics are shown in Table 1. No significant differences were observed at baseline between visits 1 and 2 in terms of lung function (spirometry), respiratory mechanics (oscillometry), and inflammation (FeNO). The same final methacholine concentration was reached on both visits for all patients, except one. In the latter, the challenge was stopped prematurely in visit 2 (at 2 instead of 8 mg/mL of methacholine) because the dyspnea Borg score rose above 7/10.

**TABLE 1:** Demographics, as well as baseline lung function.

Sample size	16		
Sex (% female)	75		
Age (years)	29.0 ± 14.5		
Ethnicity	16 Caucasians		
Number of ever smoker	0		
BMI (kg/m^2^)	26.6 ± 5.1		
Medication, number of patients	SABA	11	
	LABA	6	
	ICS^a^	12	
	LTRA	0	
TLC (L)	6.2 ± 0.8		
TLC (% of predicted value)	107.9 ± 10.9		
FRC (L)	3.1 ± 0.5		
FRC (% of predicted value)	103.9 ± 11.3		
RV (L)	1.9 ± 0.5		
RV (% of predicted value)	118.0 ± 25.1		
	**Visit 1**	**Visit 2**	***p* value**
FEV_1_ (L)	3.4 ± 0.6	3.5 ± 0.5	0.37
FEV_1_ (% of predicted value)	94.1 ± 13.5	96.2 ± 13.3	0.30
R_rs5_ (cmH_2_O·s/L)	3.9 ± 1.3	3.9 ± 1.3	0.75
R_rs19_ (cmH_2_O·s/L)	3.2 ± 0.6	3.2 ± 0.7	0.95
R_rs5‐19_ (cmH_2_O·s/L)	0.7 ± 1.1	0.7 ± 0.9	0.59
X_rs5_ (cmH_2_O·s/L)	−1.9 ± 1.3	−1.7 ± 1.0	0.34
F_res_ (Hz)	18.3 ± 8.9	18.8 ± 9.3	0.73
Ax (cmH_2_O/L)	13.6 ± 18.8	12.8 ± 15.1	0.72
FeNO (ppb)	22.2 ± 11.5	26.1 ± 15.6	0.15
Final methacholine concentration (mg/mL)	3.2 ± 4.2	2.8 ± 4.0	0.33

*Note*: Numbers are means ± SD.

Abbreviations: Ax, reactance area between 5 Hz and F_res_; BMI, body mass index; FeNO, fraction of exhaled nitric oxide; FEV_1_, forced expiratory volume in 1 s; FRC, functional residual capacity; F_res_, resonant frequency; ICS, inhaled corticosteroids; LABA, long‐acting β_2_‐agonists; LTRA, leukotriene receptor antagonists; ppb, parts per billion; R_rs19_, respiratory system resistance at 19 Hz; R_rs5_, respiratory system resistance at 5 Hz; R_rs5‐19_, R_rs5_ minus R_rs19_; RV, residual volume; SABA, short‐acting β_2_‐agonists; TLC, total lung capacity; X_rs5_, respiratory system reactance at 5 Hz.

^a^ The mean dose of ICS for nine patients was 250.0 ± 108.3 μg of fluticasone propionate equivalent. The other three patients were prescribed ICS as needed.

**FIGURE 3:**
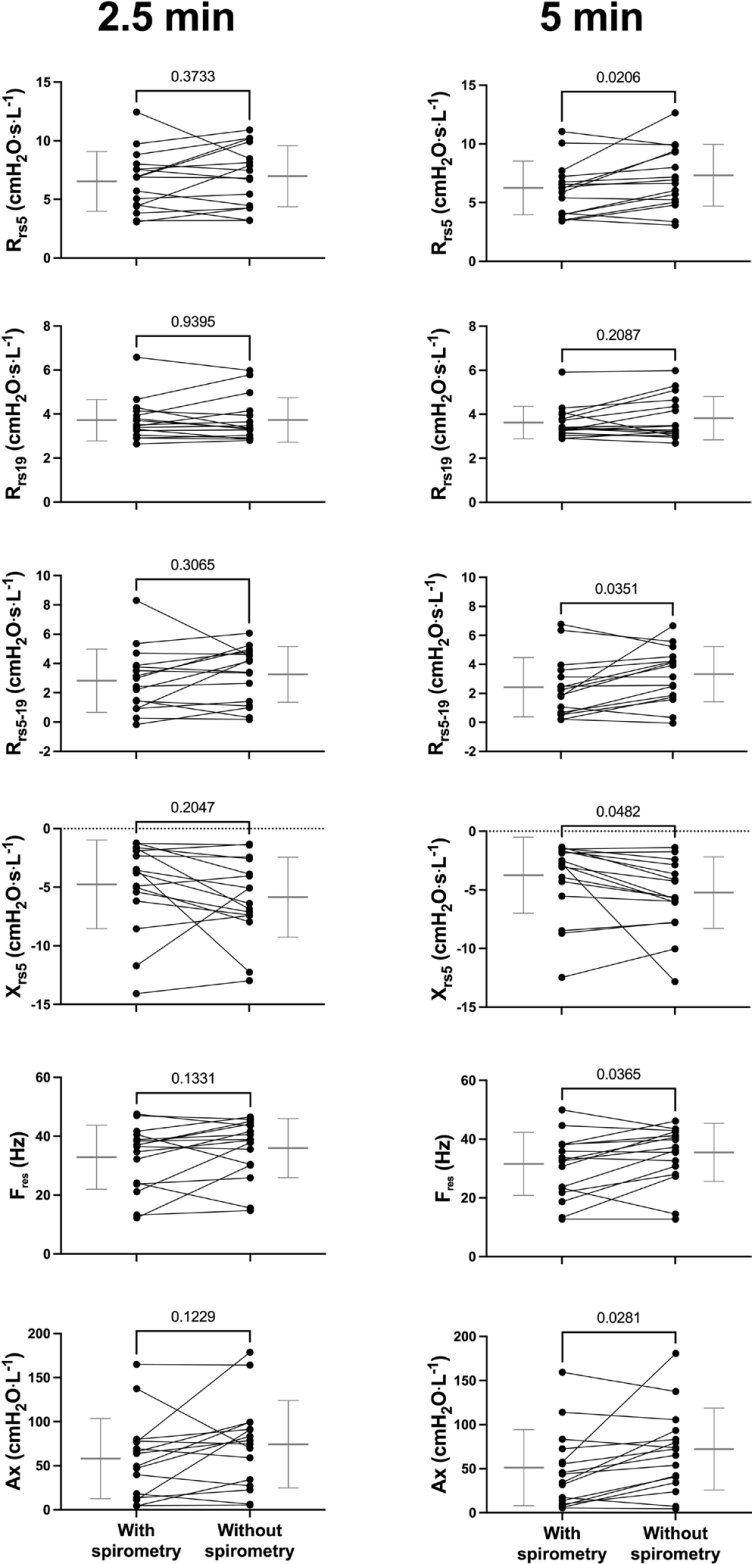
Maximal methacholine responses of all patients on visit 1, where spirometry was performed, and on visit 2, where spirometry was not performed. The results at 2.5 and 5 min after the beginning of nebulization of the final concentration are displayed on the left and right, respectively. Respiratory system resistance at 5 Hz (R_rs5_), respiratory system resistance at 19 Hz (R_rs19_), R_rs5_ minus R_rs19_ (R_rs5‐19_), respiratory system reactance at 5 Hz (X_rs5_), resonant frequency (F_res_) and reactance area (Ax) are shown from top to bottom. Statistics are based on paired t‐tests. Individual results are shown (solid circles), together with the mean ± SD (gray) of each visit. For each patient, a line is connecting the results of both visits. *n* = 16.

The oscillometric results of a representative patient are depicted in Figure 2. The maximal response for each patient, the one after the final methacholine concentration, is depicted in Figure 3. The entire concentration‐response, as in Figure 2, cannot be shown in groups because the patients were exposed to a different final concentration, as well as to a different number of concentrations. At 2.5 min after the beginning of nebulization of the final concentration, there were no differences in the oscillometric results between visits. However, at 5 min, the maximal response of all oscillometric readouts, except for R_rs19_, was greater in visit 2 (without spirometry) than in visit 1 (with spirometry). The magnitude of the changes in oscillometric readouts caused by the final concentration of methacholine compared to baseline, as well as the percentage differences between visits are also shown in Table 2.

**TABLE 2:** Changes induced by methacholine between visits.

	2.5 min				5 min			
	Visit 1	Visit 2	Delta (%)^a^	*p* value	Visit 1	Visit 2	Delta (%)^a^	*p* value
ΔR_rs5_ (cmH_2_O·s/L)	2.6 ± 2.1	3.1 ± 2.0	18.9	0.29	2.0 ± 1.7	3.2 ± 2.1	55.9	**0.012**
ΔR_rs19_ (cmH_2_O·s/L)	0.5 ± 0.6	0.5 ± 0.6	0.9	0.97	0.4 ± 0.4	0.6 ± 0.6	49.0	0.19
ΔR_rs5‐19_ (cmH_2_O·s/L)	2.1 ± 1.9	2.6 ± 1.7	23.2	0.24	1.6 ± 1.6	2.6 ± 1.7	57.5	**0.018**
ΔX_rs5_ (cmH_2_O·s/L)	−2.9 ± 3.0	−4.1 ± 3.2	41.9	0.14	−2.1 ± 2.3	−3.7 ± 2.7	75.8	**0.025**
ΔF_res_ (Hz)	14.6 ± 9.6	17.2 ± 9.2	17.6	0.31	12.3 ± 7.6	15.7 ± 9.3	28.1	0.16
ΔAx (cmH_2_O/L)	44.3 ± 36.5	61.3 ± 45.2	38.5	0.10	33.9 ± 31.4	55.9 ± 42.8	65.0	**0.018**

*Note*: Numbers are means ± SD. Bold *p* values signify that they are statistically significant.

Abbreviations: Ax, reactance area between 5 Hz and F_res_; F_res_, resonant frequency; R_rs19_, respiratory system resistance at 19 Hz; R_rs5_, respiratory system resistance at 5 Hz; R_rs5‐19_, R_rs5_ minus R_rs19_; X_rs5_, respiratory system reactance at 5 Hz.

^a^ Delta in percentage was calculated as: (value of visit 2 − value of visit 1) ÷ value of visit 1 × 100.

The concentration of methacholine that was required to result in an equivalent change in X_rs5_ between visits was then compared. For this comparison, the PC_20_ was used, except for three patients who did not reach the PC_20_. In the latter, the final concentration was used. This comparative concentration was on average 2.8 ± 4.2 mg/mL in visit 1 and did change X_rs5_ by an average of 79.1 ± 54.2%. In order to reach the same average change in X_rs5_ in visit 2, a concentration of 1.3 ± 2.0 mg/mL was required (*p* = 0.116). In terms of doubling concentrations, this change in X_rs5_ was attained by 3.7 ± 1.8 doubling concentrations in visit 1 versus 2.8 ± 1.6 doubling concentrations in visit 2 (*p* = 0.0078). This means that an equivalent percentage increase in X_rs5_ was attained with a lower number of doubling concentrations in visit 2 compared to visit 1. On an individual basis, the number of doubling concentrations to achieve the same change in X_rs5_ would have changed by −3 in two patients, by −2 in two patients, by −1 in six patients, by 0 in five patients, and by +1 in one patient in visit 2 compared to visit 1.

**FIGURE 4:**
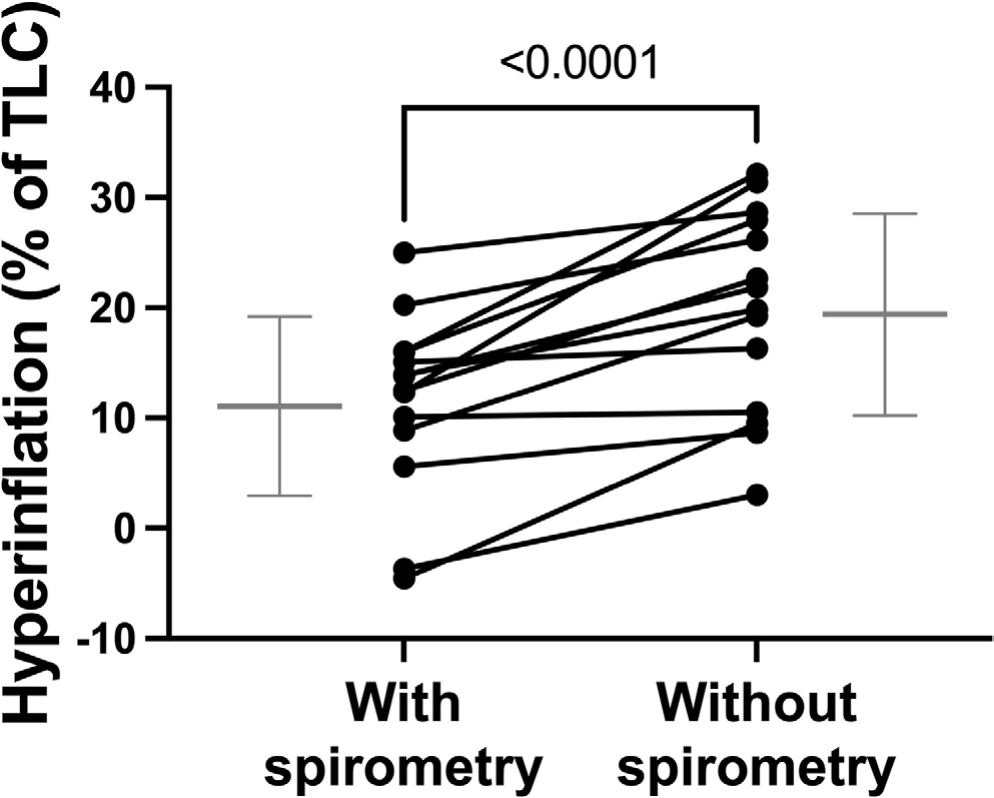
The degree of hyperinflation at the end of the methacholine challenge is compared between visits. Statistics are based on paired t‐tests. Individual results are shown (solid circles), together with the mean ± SD (gray) of each visit. For each patient, a line is connecting the results of both visits. *n* = 14.

Hyperinflation, the difference between EELV at the end of the methacholine challenge in visit 1 or 2 and FRC in visit 0 expressed in percentage of TLC, is depicted in Figure 4. One patient did not take the deep inspiration to TLC before leaving the device during the last oscillometric measurement in visit 2 (the operator forgot to repeat this instruction). As this deep inspiration was required to calculate the degree of hyperinflation, this patient was excluded from this analysis. Another patient was excluded because the last methacholine concentrations used in visit 1 were not tolerated in visit 2. This patient was excluded because the comparison of hyperinflation between visits is only valid if done at the same final concentration. The final sample size for this analysis was thus restricted to 14. The results demonstrate that hyperinflation was observed on both visits, but it was substantially greater in visit 2 than visit 1 (*p* < 0.0001). Note that two patients in visit 1 had a negative value of hyperinflation, meaning that they deflated (i.e., their EELV decreased) during the methacholine challenge.

## 4 DISCUSSION

No previous studies have directly compared the oscillometric response to methacholine with and without concomitant spirometry. This comparison has previously been called for (Donohue & Kaminsky, 2024), including in current guidelines (King et al., 2020). The present study demonstrated that the deep inspirations required in spirometry to track lung function during a methacholine challenge attenuate the oscillometric response.

Many investigators have evaluated the methacholine response by using both spirometry and oscillometry during the same challenge (Bailly et al., 2011; Broeders et al., 2005; Corral‐Blanco et al., 2024; Jara‐Gutierrez et al., 2019; Kim et al., 2009; Mansur et al., 2008; Naji et al., 2013; Nazemiyah et al., 2021; Petak et al., 2012; Schulze et al., 2012; Short et al., 2015; Urbankowski & Przybylowski, 2021; Vink et al., 2003; Yoon et al., 2014). Others have used oscillometry alone; i.e., without comparing it with the combined use of oscillometry and spirometry (Jee et al., 2010; Kalliola et al., 2014; Klug & Bisgaard, 1997). The results indicated that oscillometry is either equivalent or superior to monitoring the methacholine response or detecting hyperresponsiveness (i.e., a decline in FEV_1_ of more than 20% with a concentration of methacholine inferior to 4 mg/mL). Since oscillometry is less demanding for both the patient and the operator, these studies already suggested that oscillometry should be the technique of predilection for methacholine testing.

Some advantages of oscillometry may also be potentiated without concomitant spirometry. Indeed, excluding forceful breathing maneuvers should decrease the risk of exposure to airborne pathogens (and respiratory infections) for the operator and other health practitioners working at or near lung physiology labs (Gupta et al., 2021; Kouri et al., 2020; Lundblad & Chow, 2020). Oscillometry alone is also likely to decrease the concentration of methacholine needed to report a positive response, since deep inspirations, such as the ones required for measuring FEV_1_, attenuate the methacholine response (Allen et al., 2008; Chapman et al., 2011; Duggan et al., 1990; Hida et al., 1984; Kapsali et al., 2000; King et al., 2001; Malmberg et al., 1993; Nadel & Tierney, 1961; Salerno et al., 2005; Scichilone et al., 2001; Skloot & Togias, 2003). Although long suspected (Petak et al., 2012), this latter benefit had never been directly quantified before. Using oscillometry alone may also improve the diagnosis of asthma by increasing the number of positive test results, depending on the cut‐offs chosen for the oscillometric readouts.

The present study was specifically designed to compare the oscillometric response to methacholine with or without concomitant spirometry in a group of very mild to mild asthmatics. The results demonstrated that omitting the spirometric measurements enhances, at least on average, the oscillometric response to methacholine, which confirms long‐held suspicions (Petak et al., 2012). This was shown for all oscillometric readouts, except for R_rs19_. It is worth mentioning that R_rs19_ changed relatively less compared to the other oscillometric readouts (Table 2), probably due, at least in part in this study, to the low methacholine concentrations used with asthmatics. We have also previously demonstrated, in both healthy and asthmatic individuals, that the effect of methacholine during a serial challenge with incremental concentrations is cumulative for oscillometric readouts appraising the lung and small airways (R_rs5_, R_rs5‐19_, X_rs5_, F_res_, Ax) but not for readouts appraising exclusively large airways (R_rs19_) (Henry et al., 2023), which may also account for the lack of difference in R_rs19_ between visits in the present study. Previous studies have also documented that large airways are less responsive to the bronchodilator effect of deep inspirations than small airways (Brown et al., 2001; Marchal et al., 2002), which may be another reason why the changes in R_rs19_ were not different between visits.

Importantly, although an equivalent change in X_rs5_ was attained with a lower number of doubling concentrations in visit 2 than visit 1 (*p* = 0.0078), this difference did not represent one doubling concentration. This means that the final concentration used, as well as the duration of the methacholine test, would only be reduced in a fraction of patients. The effect size reported in the present study is therefore not as pronounced as anticipated. In fact, there were no significant differences between visits at 2.5 min (Figure 3), corresponding to the time point after the final concentration before spirometric maneuvers. This means that all deep inspirations during the preceding concentrations did not influence the oscillometric response to the final concentration. Yet, it is also understood that at concentrations lower than the PC_20_, the response is oftentimes weak or nonexistent, which likely foreclosed the bronchodilator effect of deep inspirations and perhaps explains the lack of difference between visits up to that point. Another possibility contributing to both the difference at 5 min and the lack of difference at 2.5 min is that the deep inspirations used to monitor the response to the preceding doses may have enhanced the bronchodilator effect of the deep inspirations used to monitor the response to the final dose. Indeed, Crimi and coworkers have previously demonstrated that the bronchoprotective effect of a series of deep inspirations was related to the fact that it potentiates the bronchodilator effect of a subsequent deep inspiration (Crimi et al., 2002).

A striking, and more surprising, difference between visits was the level of hyperinflation achieved at the end of the methacholine challenge. Although hyperinflation during a methacholine challenge is a phenomenon understudied, it is consistently reported when investigated (Eddy et al., 2020; Geier et al., 2019; Henry et al., 2023; Smith et al., 2020). Herein, hyperinflation was observed on both visits but was greater in visit 2 (19.9 ± 9.2% of TLC) compared to visit 1 (11.5 ± 8.1% of TLC). This difference is substantial. For a patient with a TLC of 8 L who is normally breathing at an EELV (or FRC) of 4 L, for example, this would mean breathing at 5.6 L in visit 2 instead of 4.9 L in visit 1. It confirmed, once again, that the methacholine response was greater without spirometric measurements.

Hyperinflation is obviously an important confounding factor because respiratory mechanics, as well as the methacholine‐induced changes in respiratory mechanics, are strongly related to lung volume (Briscoe & Dubois, 1958; Ding et al., 1987; Gobbi et al., 2024). In fact, increasing EELV in healthy volunteers exerts a greater attenuating effect on the methacholine response than increasing the dynamic swings in tidal volume (Gobbi et al., 2024). Breathing at a higher lung volume decreases resistance because it stretches the airways open, and it also typically decreases elastance (and therefore increases reactance) because it recruits small airways and volume. The greater hyperinflation in visit 2 may thus explain why the size of the reported effect was not as large as anticipated, or why there were still no differences between visits at 2.5 min after inhaling the final methacholine concentration.

Together, these suggest that the greater oscillometric response in visit 2 versus visit 1 may have been amplified if one had been controlling for lung volume. Since hyperinflation is unconscious, its blunting effect on the methacholine response will remain a limitation, probably carrying more weight in oscillometry than spirometry. Although not recommended in current guidelines (King et al., 2020), we suggest that a deep inspiration to TLC at the last oscillometric measurement (before leaving the mouthpiece) should be incorporated in routine testing, especially during a methacholine challenge, so that the volume at which the patient is breathing could at least be reported. Notably, experimental data on isolated bronchi also demonstrated that hyperinflation blunted the bronchodilator response to deep inspirations (Cairncross et al., 2018). Since hyperinflation was observed in visit 1, it may have diminished the differences between visits 1 and 2 and contributed to the small effect size reported in the present study.

Another reason why the effect size was small may be related to the studied population. Indeed, asthmatic patients are known to exhibit a weaker bronchodilator response to deep inspirations (Allen et al., 2008; Black et al., 2004; Burns et al., 1985; Fish et al., 1981; Jensen et al., 2001; Lim et al., 1987; Salome et al., 2003; Scichilone et al., 2001; Skloot et al., 1995; Slats et al., 2007; Wheatley et al., 1989), thereby explaining the small effect of spirometry on the oscillometric response in the present study. The differences between visits may have also been mitigated in asthmatic patients because of their faster rate of re‐narrowing following the bronchodilation caused by the deep inspiration (Brown et al., 2001; Jackson et al., 2004; Jensen et al., 2001; Salome et al., 2003). It is also worth mentioning that the bronchodilator response to deep inspirations in asthma is related to disease severity (Allen et al., 2008). In fact, the response can even be inversed in more severe asthma cases, where a deep inspiration may cause a bronchoconstriction (Fish et al., 1977; Lim et al., 1989). The reported effect size in the present study may thus vary according to the population studied, being possibly greater in non‐asthmatic individuals and possibly smaller, perhaps nonexistent, in more severe asthmatic patients. Other studies will be needed.

### 4.1 Conclusion

The oscillometric response to methacholine is significantly attenuated by concomitant spirometry. Using oscillometry alone also reduces the number of doubling concentrations needed to reach an equivalent response. Yet, because hyperinflation during the challenge is furthered in the absence of periodic forceful spirometric maneuvers starting from a full deep inspiration, the size of this effect is small. By no means would the results of the present study discourage the use of oscillometry and spirometry in the same challenge. However, if the two techniques are to be used individually in clinical settings, it is argued that oscillometry supplants spirometry for methacholine testing, not only for people unable to perform spirometry but for everyone. This is partially based on the results of the present study, showing that the impact on clinical outcome would be minimal and it would hasten the test for a significant proportion of patients by reducing the number of methacholine doses, but also because oscillometry is easier (requiring no forceful expiratory maneuver starting from a full deep inspiration) than spirometry.

## AUTHOR CONTRIBUTIONS

YB had full access to all of the data in the study and takes responsibility for the integrity of the data and the accuracy of the data analysis. CH, MB, MÈB, and AC contributed substantially to the study design, data analysis, and interpretation, and the writing of the manuscript.

## FUNDING INFORMATION

This research was funded by: The Canadian Institutes of Health Research (CIHR, 508356‐202209PJT); and the Natural Sciences and Engineering Research Council of Canada (NSERC, RGPIN‐2020‐06355).

## CONFLICT OF INTEREST STATEMENT

AC is holding or has previously received grants or contracts from AstraZeneca, Regeneron, and Glaxo SmithKline, has previously received consulting fees or honoraria from AstraZeneca, Sanofi, Glaxo SmithKline, Valeo, and Covis, and has also served on advisory boards for AstraZeneca and Sanofi. CH, MB, MÈB, and YB have no conflicts of interest.

## ETHICS STATEMENT

This study has obtained ethics approval from the *Institut Universitaire de Cardiologie et de Pneumologie de Québec* review board (protocol 21001), and all volunteers gave written informed consent.

## DISCLAIMERS

The content of this manuscript is solely the authors' responsibility and does not necessarily represent the official views of the funding agencies and the *Université Laval*.

## Supporting information


Data S1.

## Data Availability

The datasets used and analyzed during the current study are available from the corresponding author on reasonable request.
