# Supplementary material for: Oscillometry with or without spirometry for methacholine testing
Source: Physiol Rep. 2025 May 22;13(10):e70387. doi: 10.14814/phy2.70387 (PMC12098963; doi:10.14814/phy2.70387)
Supplement: Supplementary file 1 — Data S1. [file PHY2-13-e70387-s001.docx]

**INCLUSION CRITERIA**

1. 18 years and older.
2. Body mass index (BMI) less than 40 kg/m^2^
3. Free of respiratory infections for at least 1 month prior to each visit
4. Lifetime non-smokers
5. FEV_1_ above 60% of predicted
6. Classified as asthmatics in Steps 1 to 3 according to GINA guidelines^1^.
7. Able to reach the concentration of 0.06 mg/mL or more during the methacholine bronchoprovocation.

**EXCLUSION CRITERIA**

1. Unstable diabetes (Type I or II)
2. Any cardiovascular disease
3. Aortic aneurysm
4. Unstable hypertension
5. Pacemaker or any implanted electronic devices
6. History of recurrent lower respiratory tract infections
7. Any significant concomitant illness or injury that would interfere with the subject’s participation in the study.
8. Use of β-blockers or any medication that may interfere with heart rate.
9. Use of any concomitant medication that, in the opinion of the investigator, may affect the outcome of the study.
10. Use of inhaled anticholinergics or combination products (Atrovent, Combivent, Spiriva).
11. Use of anticholinergic-containing drugs.
12. Systemic immuno-suppressive or immuno-modulatory drug therapy (systemic corticosteroids tablets, suspension or injection), cyclophosphamide, methotrexate, cyclosporine, etc.) taken within 6 weeks prior to study entry.
13. Use of products containing caffeine or medications for 4 h, short-acting inhaled bronchodilators for 8 h, or long-acting inhaled bronchodilators for 24 h prior to methacholine challenge.
14. Pregnancy and nursing mother
15. Any condition that makes the subject inappropriate for the scheduled procedures based on the judgment of the respirologist, including capacity to provide informed consent and complete the study, as well as the inability to perform spirometry with acceptable quality.
16. Lung disease other than mild to moderate asthma
17. Severe asthma; *i.e.*, classified as Step 4 or 5 of GINA guidelines^1^
18. Baseline FEV_1_ <60% of predicted
19. Unstable asthma defined as ≥ 3 hospitalizations for asthma exacerbation in the previous year, a history of life-threatening asthma based on previous intubation, ICU admission for asthma in the previous 24 months, or ≥ 4 pulses of oral steroids in the past 12 months.
20. Any respiratory condition other than asthma that may interfere with the tests (*e.g.*, heart failure, infection, coronary arterial disease, COPD, interstitial pulmonary disease or other cardiopulmonary disorders causing dyspnea).
21. PC_20_ below 0.06 mg/mL on the first visit

**REFERENCES**

1 Global Initiative for Asthma. 2021. Global strategy for asthma management and prevention, 2021. Available from: <http://www.ginasthma.org/reports>. Accessed: March 2022.
